# Halogen effects on the electronic and optical properties of Au_13_ nanoclusters†

**DOI:** 10.1039/d0na00662a

**Published:** 2020-08-31

**Authors:** Ze-Hua Gao, Jia Dong, Qian-Fan Zhang, Lai-Sheng Wang

**Affiliations:** Department of Chemistry, Brown University Providence RI 02912 USA lai-sheng_wang@brown.edu

## Abstract

We report an experimental and theoretical investigation of the electronic and optical properties of a series of icosahedral Au_13_ nanoclusters, protected using different halogen ligands (Cl, Br, and I), as well as 1,2-bis(diphenylphosphino)ethane (dppe) ligands. All three clusters are comprised of the same Au_13_ kernel with two halogens coordinated to the poles of the icosahedral cluster along with five dppe ligands. UV-vis absorption spectra indicate a systematic red shift from Cl to Br to I, as well as a sudden enhancement of the second excitonic peak for the I-coordinated cluster. Density functional theory (DFT) calculations suggest that all clusters possess a wide HOMO–LUMO energy gap of ∼1.79 eV and are used to assign the first two excitonic bands. Frontier orbital analyses reveal several HOMO → LUMO transitions involving halogen-to-metal charge transfers. For the I-coordinated cluster, more complicated I-to-metal charge transfers give rise to different excitation features observed experimentally. The current findings show that halogen ligands play important roles in the electronic structures of gold clusters and can be utilized to tune the optical properties of the clusters.

## Abstract 2

We have demonstrated that halogens can greatly influence the electronic and optical properties of Au_13_ nanoclusters.

## Graphical abstract

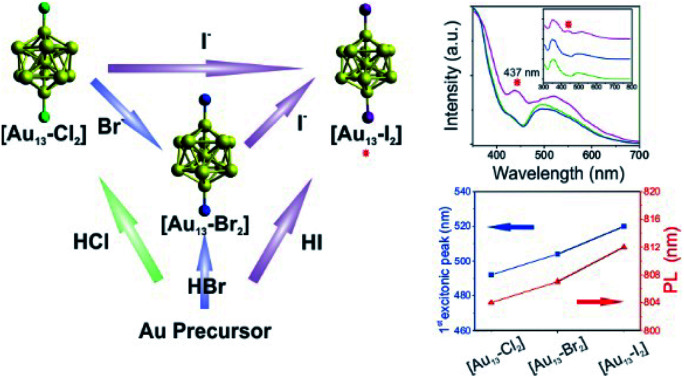

## 1. Introduction

Even though bulk gold displays a beautiful golden color, the photophysical properties of nanogold are size-dependent due to quantum confinement of the conduction electrons.^1,2^ Specifically, gold nanoclusters with dimensions in the subnanometer size regime possess unique molecular characteristics, allowing for precise structure determination and application in photoluminescence (PL),^3–6^ catalysis,^7–10^ and biomedicine.^11,12^ With the availability of single crystal X-ray structures,^13,14^ extensive attention has been paid to the role of ligands in the syntheses and photophysical properties of gold nanoclusters with different nuclearities.^15,16^

To obtain novel gold clusters with different photophysical properties, organic ligands such as thiols, phosphines and alkynes have been widely exploited in the syntheses of gold nanoclusters (Au_*n*_) such as Au_8_,^17,18^ Au_9_,^19^ Au_11_,^20,21^ Au_13_,^22–27^ Au_18_,^27,28^ Au_20_,^29,30^ Au_22_,^31,32^ Au_23_,^33^ Au_24_,^34^ Au_25_,^35,36^ Au_32_,^37^ Au_37_,^38^ Au_44_,^39^ Au_52_,^40^ Au_55_,^41^ and Au_102_.^13^ Among all of the gold nanoclusters that have been structurally characterized, icosahedral Au_13_ nanoclusters have been more extensively studied due to their highly symmetrical structure and prominent photoluminescence properties. For example, bis(diphenylphosphino) ligand (Ph_2_P–(CH_2_)*m*–PPh_2_, *m* = 2, 3, and 4) protected [Au_13_P_10_Cl_2_]^3+^ and [Au_13_P_8_Cl_4_]^+^ clusters have been reported to exhibit near infrared PL by Konishi *et al.*^24,25^ In addition, an N-heterocyclic carbene (NHC) was employed by Crudden and co-workers to synthesize [Au_13_(NHC)_9_Cl_3_]^2+^, which possessed enhanced stability and exceptionally high PL quantum yield (PLQY) (16.0%).^42^ Icosahedral Au_13_ was also identified as kernels in the structures of larger gold clusters such as Au_25_, Au_37_ and Au_60_.^23^ Furthermore, density functional theory (DFT) calculations indicated that a metallic (Au_13_)^5+^ core can be viewed as a superatom with a closed electron configuration of (1s)^2^(1p)^6^.^43^ Despite extensive studies, the effects of halide ligands on the PL or electronic structures of Au_13_ clusters have not been examined.

However, the Au–halogen interactions in a series of Au compounds in the gas phase, including [AuX_2_]^−^ (X = halogens) and [XAuCN]^−^, have been investigated by photoelectron spectroscopy previously.^44,45^ Periodic trends were found in the bond lengths and covalent nature of the Au–X bond.^44^ In addition, halogens have been found to take an active role in the synthesis of nanoparticles (NPs) for morphology control^46–48^ and PL.^49,50^ Although there have been sophisticated characterization methods and theoretical calculations, it is a challenge to understand halogen effects due to their large size and the inhomogeneity of NPs. Therefore, small nanoclusters with an atomically precise structure and molecule-like size are more suitable systems to study the halogen effects. Relatively few studies have been carried out on gold clusters with different halogen ligands. The transformation of halogen-stabilized Au_8_ to Au_11_ was investigated by Yam *et al.*^19^ Wang and co-workers determined the structure of a large Au_80_Ag_30_ bimetallic nanocluster and suggested that the halide ions functioned more than counterions in the formation of large clusters.^51^ To the best of our knowledge, there has been no research regarding the influence of the electronic structure of gold nanoclusters by halogens. Chloride is usually the *de facto* ligand in gold cluster syntheses, probably because of the easy availability of the ClAuPPh_3_ starting material. Methods to directly synthesize bromide- or iodide-coordinated gold clusters are not usually achievable. Furthermore, the intrinsic photophysical properties of gold clusters and their influence by large organic ligands instead of halogens have been the focus.

In the current article we report a systematic study about halogen effects on Au_13_ nanoclusters. We used both direct syntheses and halide exchanges to obtain three halogen-coordinated clusters, [Au_13_(dppe)_5_X_2_]X_3_ (X = Cl, Br, and I) (abbreviated as [Au_13_–X_2_] hereafter). Both methods are facile enough to obtain [Au_13_–Br_2_] and [Au_13_–I_2_]. All three clusters have been fully characterized by electrospray ionization mass spectroscopy (ESI-MS) and NMR spectroscopy. Both experimental and theoretical results show that the [Au_13_–Br_2_] and [Au_13_–I_2_] clusters retain the same icosahedral Au_13_ kernel as the original [Au_13_–Cl_2_] cluster. The photophysical properties of the [Au_13_–X_2_] clusters are systematically studied by UV-vis absorption spectroscopy, PL and photoluminescence excitation spectroscopy (PLE). We have observed that both the first excitonic peak and the emission peak show a consistent red shift from X = Cl to I. However, we observed an unusual PLE spectrum for the I-ligated cluster. Theoretical calculations confirmed that the p-orbitals of the halogen ligands contribute to the HOMO → LUMO transition. Additional non-radiative transitions in the [Au_13_–I_2_] cluster resulted in lower PL intensities. These findings provide further insights into the ligand-to-metal charge transfer process. Finally, the synthetic methods and the knowledge about halogen–gold cluster interactions will be useful to guide future designs for halogen-ligated gold clusters.

## 2. Results and discussion

### 2.1 The syntheses of [Au_13_–X_2_] (X = Cl, Br, and I)

**Fig. 1:**
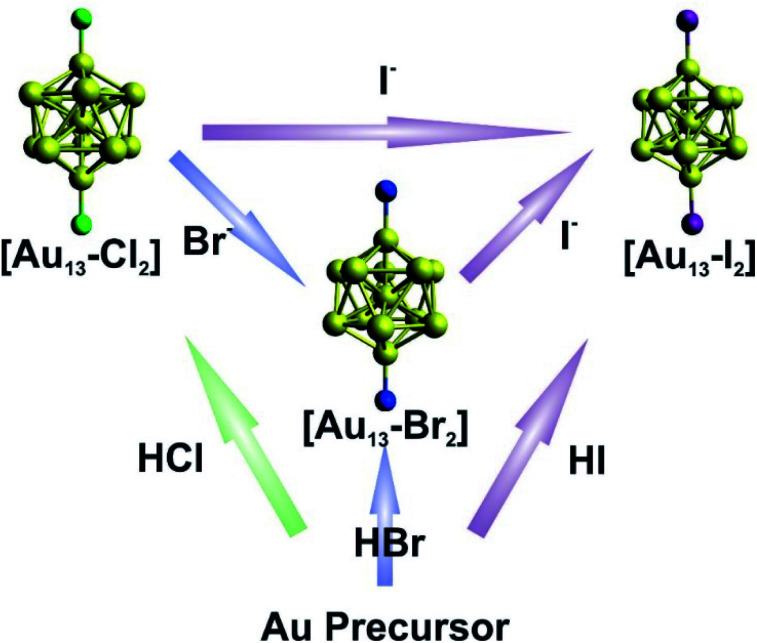
Schematic illustration of the direct synthesis (bottom up) and halide exchange (left to right) for [Au_13_–X_2_].

The syntheses of the three [Au_13_–X_2_] clusters were carried out under ambient conditions. Detailed synthetic methods are described in the Experimental section. The [Au_13_–X_2_] clusters could all be synthesized by an acid-assisted two-step method (Fig. 1) according to a previously reported method with some modifications (see the Experimental section).^25^ Halide exchanges could also be used to obtain the Br- or I-ligated clusters (Fig. 1). Briefly, a CH_2_Cl_2_ solution of Au_2_(dppe)Cl_2_ was reduced using NaBH_4_. After purification, halogen acids were added and the corresponding [Au_13_–X_2_] clusters were obtained. It should be noted that light shading is essential for the successful synthesis of the I-coordinated cluster. The halide exchange method was also performed between clusters as shown in the top of Fig. 1. By introducing stoichiometric amounts of KBr or KI salts into a methanol solution of [Au_13_–Cl_2_], exchange reactions rapidly happened with significant color change. We should point out that the reactions only occurred from Cl to Br to I, and the reverse reactions were not observed. This result indicated that the Au–X bond strength increases from Cl to I, due to the increased Au–X covalency.^44,45^

### 2.2 Composition and structural characterization using ESI-MS and NMR spectroscopy

**Fig. 2:**
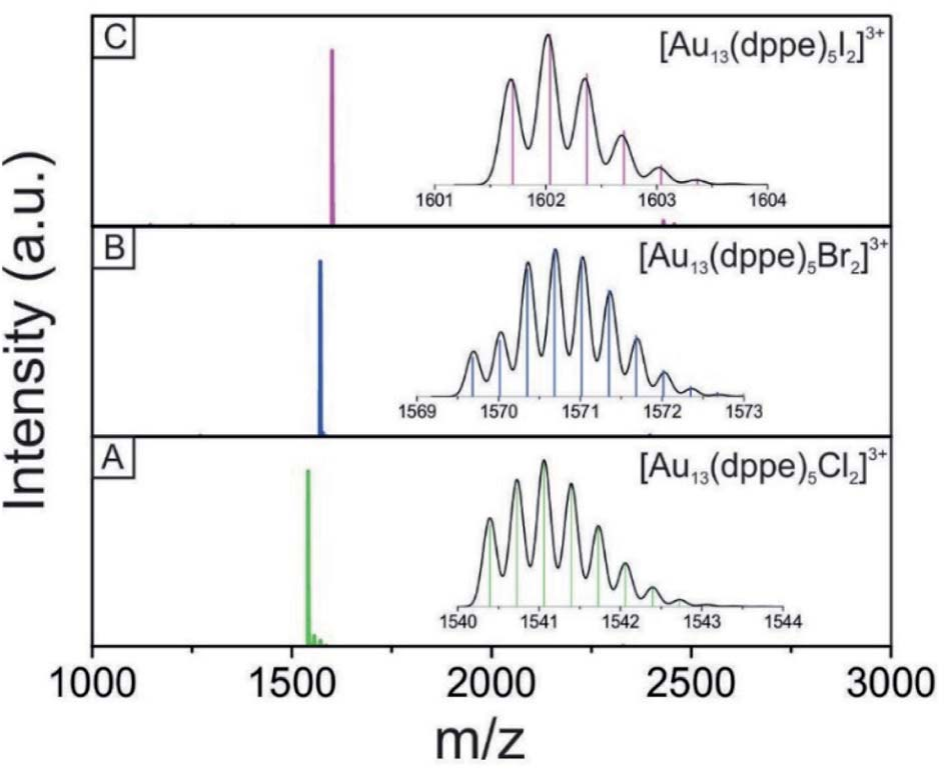
Positive-mode ESI mass spectra of the triply charged [Au_13_–X_2_]^3+^ cluster ions. (A) [Au_13_–Cl_2_], (B) [Au_13_–Br_2_], and (C) [Au_13_–I_2_]. The inset in the panel shows the simulated isotopic distribution pattern (black curve) for each cluster.

We used electrospray mass spectrometry and NMR spectroscopy to verify the composition and purity of the [Au_13_–X_2_] clusters after the purification process. The positive-ion ESI mass spectra of all three species exhibited a single peak in the mass-charge ratio range from 0 to 8000 (Fig. 2 and S1†). Comparison with the simulated isotopic distributions (insets in Fig. 2) confirmed the triply charged [Au_13_(dppe)_5_X_2_]^3+^ cation in each case. Therefore, with the same charge state, gold nuclearity and diphosphine ligands, these three species showed good composition consistency in the mass spectroscopy.

**Fig. 3:**
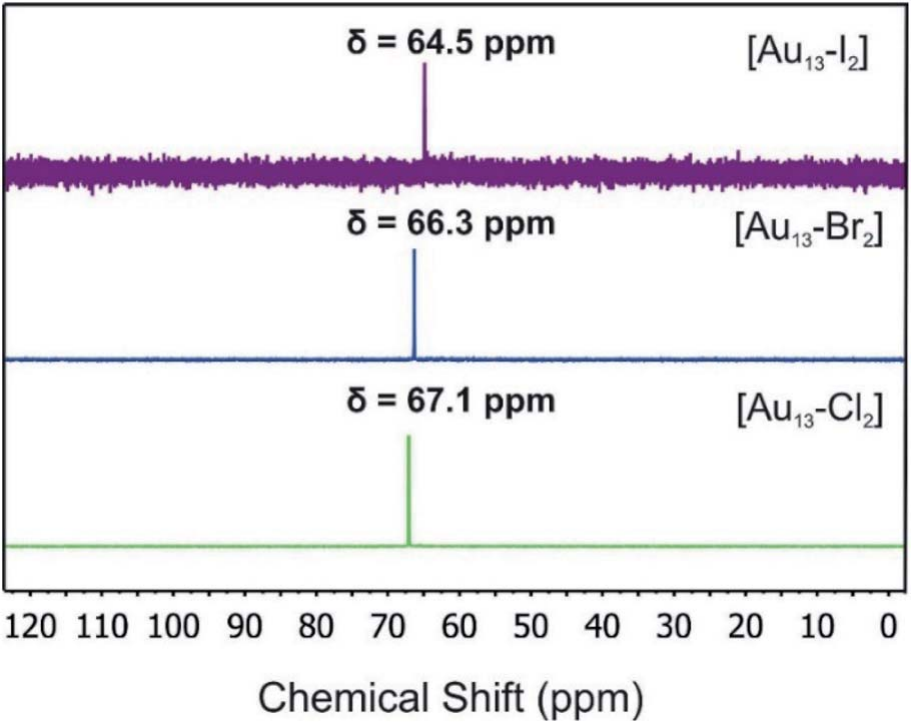
^31^P-NMR spectra of the [Au_13_–X_2_] clusters.

The ^31^P-NMR spectra of all three clusters are shown in Fig. 3. The existence of a single peak indicated the similar chemical environments of all the phosphorus atoms in the three clusters. The heavier halogen atoms shifted the peak upfield. Both the ESI mass spectra and the ^31^P NMR data showed that the three [Au_13_–X_2_] clusters we obtained were pure enough for further spectroscopy analyses. Furthermore, the ^1^H-NMR results (Fig. S2 and S5†) showed a H_aromatic_/H_alkyl_ ratio of 5/1 in good agreement with that in dppe ligands. Overall, the ^31^P and ^1^H-NMR data confirmed the highly symmetric structures and identical ligand configurations in all three [Au_13_–X_2_] clusters.

**Fig. 4:**
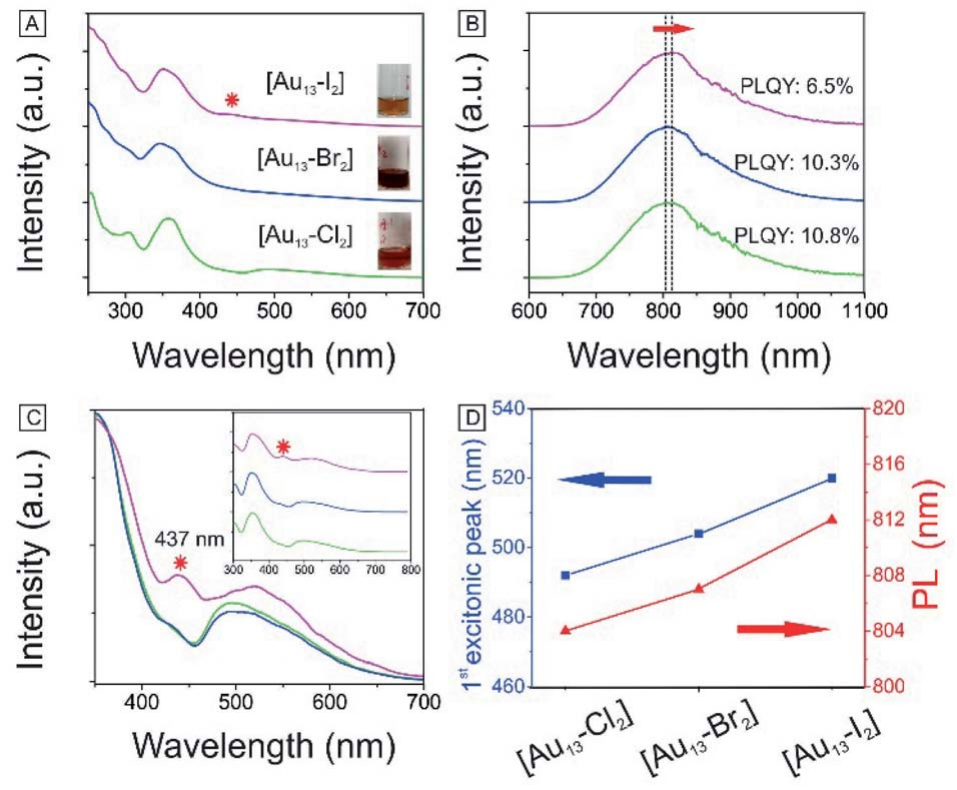
Photophysical properties of [Au_13_–X_2_] in CH_3_OH: (A) absorption spectra. Inset: Pictures of cluster solutions. Red star: absorption feature at 437 nm. (B) PL spectra and absolute PLQYs. (C) PLE spectra (*λ* = 800 nm). Red star: the second excitation feature at 437 nm. (D) Evolutions of the first excitonic peak and PL position for the three clusters. For A, B and C, [Au_13_–Cl_2_] (bottom), [Au_13_–Br_2_] (middle), and [Au_13_–I_2_] (top).

We have also obtained the absorption and PL emission for the [Au_13_–X_2_] clusters (Fig. 4A and B). The spectra of the three clusters are similar. Both the absorption and emission spectra for [Au_13_–Cl_2_] are consistent with those reported previously by Konish *et al.*^24^ The UV-Vis and PL results again confirm that the as-synthesized [Au_13_–Br_2_] and [Au_13_–I_2_] clusters possess the same structure as [Au_13_–Cl_2_], which was known to adopt an icosahedral Au_13_ core with the two chlorine atoms coordinated to the two polar gold atoms and the five bidentate dppe ligands coordinated to the ten equatorial gold atoms.

### 2.3 Photophysical properties of the [Au_13_–X_2_] clusters

There are subtle differences in the UV-vis absorption and emission spectra of the three clusters due to the halogen effects (Fig. 4A and B). Although the colors of the three cluster solutions changed from red ([Au_13_–Cl_2_]) to dark red ([Au_13_–Br_2_]) to brown ([Au_13_–I_2_]) (inset in Fig. 4A), their absorption features are similar. The corresponding PL peaks showed a slight red shift from 804 nm to 814 nm and the absolute PLQYs dropped from 10.8% to 6.5% (Fig. 4B). The PL intensities are stronger than those reported previously for gold clusters.^5,17–20^ We further employed PLE spectroscopy to obtain a higher signal-to-noise ratio for the band features of the [Au_13_–X_2_] clusters, as shown in Fig. 4C. The PLE spectra resembled the absorption spectra, but provided much clearer details about the electronic transitions responsible for the PL. The PLE spectra of [Au_13_–Cl_2_] and [Au_13_–Br_2_] are similar, but that of [Au_13_–I_2_] seems quite different. The first excitonic peaks of the clusters red-shifted from 491 nm for [Au_13_–Cl_2_] to 520 nm for [Au_13_–I_2_]. The red shifts in the PLE and PL spectra are due to a decrease in the bandgaps of the clusters. It should be pointed out that these changes in optical properties are more pronounced compared with the influence of the phosphine ligand structures on the Au_13_ clusters reported by Konishi *et al.*^3,24^ Their experiments on different phosphine ligands showed no significant changes in the UV-vis and PL spectra, which led to the conclusion that the optical properties of the gold clusters were mainly influenced by the cluster nuclearity, not so much by the coordinated ligands. The current study illustrated that the coordinated halogens did have strong influence on the photophysical properties, probably due to the strong Au–halogen electronic interactions.

It is interesting to note that the red shifts of the PLE and PL features (Fig. 4D) by tuning the halogens are in agreement with those observed in halide perovskites.^52^ In addition to the red shift, we noticed the appearance of an absorption band at 437 nm for [Au_13_–I_2_] in the UV-vis absorption spectrum, as marked by * in Fig. 4A. This feature is much more pronounced in the PLE spectra (Fig. 4C). A similar second excitonic feature also seemed to exist in the [Au_13_–Cl_2_] and [Au_13_–Br_2_] PLE spectra, albeit with much lower relative intensities.

### 2.4 The optimized structures of the [Au_13_–X_2_] clusters

**Fig. 5:**
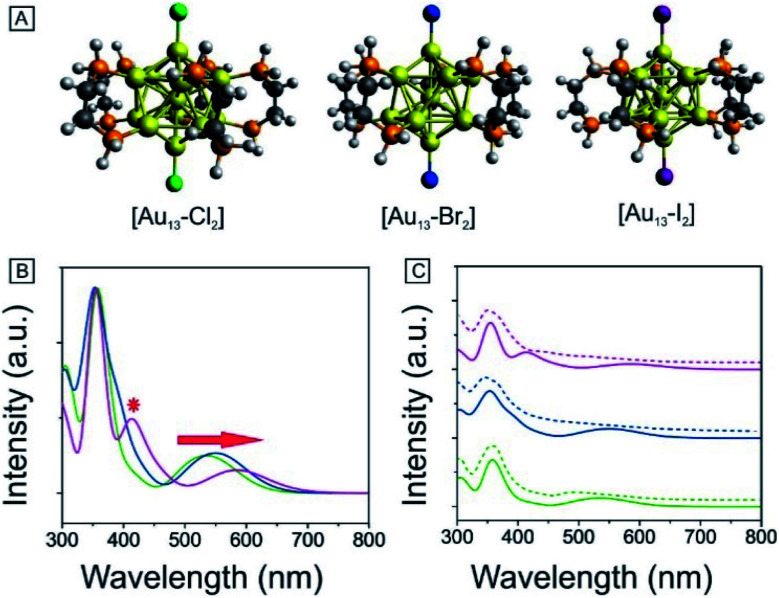
(A) Optimized structures of [Au_13_–X_2_]. (B) Calculated optical absorption spectra of [Au_13_–X_2_]. (C) Comparison of the experimental (dash line) and theoretical absorption spectra (solid line). [Au_13_–Cl_2_] (bottom, green), [Au_13_–Br_2_] (middle, blue), and [Au_13_–I_2_] (top, purple).

To obtain further insights into the electronic and optical properties of the different halogen Au_13_ clusters, we performed DFT calculations. The [Au_13_(PH_2_CH_2_CH_2_PH_2_)_5_X_2_]^3+^ clusters were modeled by replacing the phenyl rings with hydrogen atoms to simplify the calculations. All the structures were fully optimized as shown in Fig. 5A. The Au–X bond length increased from 2.335 Å for Cl to 2.629 Å for I (Table 1), consistent with the previously reported Au–X bond lengths for halide–Au(i) complexes.^44,53^ The average Au–Au distances were nearly identical for the three clusters as summarized in Tables 1 and S1,† indicating the stability of the Au_13_ core.

**Table 1:** Geometrical parameters of the ground-state structures of [Au_13_–X_2_] and comparison of DFT-predicted HOMO–LUMO gaps with the observed optical gaps

[Au_13_–X_2_]	Au–X (Å)	Average Au–Au (Å)	Optical gap (eV)	Calculated gap (eV)
Cl	2.335	2.948	1.90	1.94
Br	2.435	2.949	1.85	1.89
I	2.629	2.949	1.79	1.80

### 2.5 The electronic structure of the [Au_13_–X_2_] clusters

**Fig. 6:**
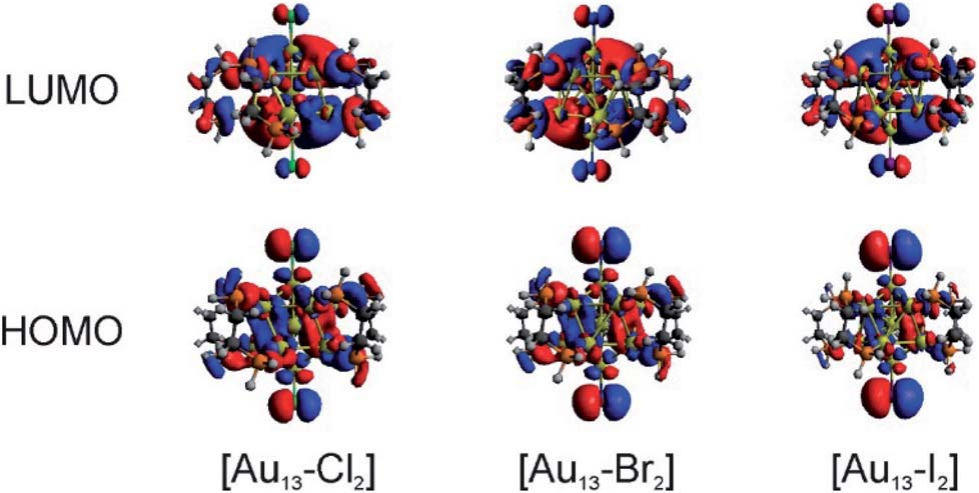
Pictures of the HOMOs and LUMOs for the [Au_13_–X_2_] clusters.

We conducted TD-DFT analyses for comparison with the absorption spectra. As shown in Fig. 5B and S11,† the theoretical absorption spectra were in good agreement with the experimental data. In particular for the [Au_13_–I_2_] cluster, the calculations revealed that the three bands located at 370 nm, 413 nm, and 600 nm were in excellent agreement with the three bands in both the absorption and PLE spectra (Fig. S11†). The lowest energy optical transition should come from the HOMO → LUMO transition. The experimental optical gaps were determined (see Fig. S8–S10†) to decrease from 1.9 eV for Cl to 1.85 eV for Br to 1.79 eV for I. At the same time, time-dependent UV-vis absorption spectra were obtained for all three clusters (Fig. S6†), and they indicated that these gaps may not be affected by temperature. The experimental energy gaps are reproduced well from the theoretical HOMO–LUMO gaps, as compared in Table 1. The 1.9 eV gap for [Au_13_–Cl_2_] has been confirmed by the result from Zhang *et al.*^53^ The large energy gaps reflect the expected electronic stability of the Au_13_ core. To understand the electronic interactions between halogens and the Au_13_ core, we show the pictures of the HOMOs and LUMOs for the three clusters in Fig. 6. The HOMOs for the three clusters are mainly derived from the p orbitals of the halogens, while the LUMOs are mainly delocalized orbitals on the Au_13_ cores. The frontier orbitals explain why the halogens have significant influences on the electronic and optical properties of the [Au_13_–X_2_] clusters, whereas the diphosphine ligands have minimal effects. The negligible influence of phosphine ligands on absorption spectra agrees well with what was observed by Konishi *et al.*^24^

Therefore, the HOMO → LUMO transitions in the [Au_13_–X_2_] clusters correspond to ligand-to-metal charge transfer (LMCT). The LMCT process was studied by Jin and co-workers on thiolate-ligated Au_25_(SR)_18_ clusters.^4^ They found that by strengthening the electron donating capability of the capped ligands, the PL intensity or the degree of LMCT of gold clusters was enhanced. However, from chlorine to iodine, the stronger electron donating capability from the halogen ligands seemed to result in lower PL intensities. The theoretical calculations provided some hints about the lower PL intensity in the [Au_13_–I_2_] cluster. The higher intensity of the second main excitonic peak for the [Au_13_–I_2_] cluster indicated a stronger electronic excitation at this wavelength. To obtain the components in the second main excitonic peak, we considered the orbital transitions related to the 413 nm peak in the calculated absorption spectra. As shown in Fig. S12–S14,† all these peaks contained multiple HOMOs and LUMOs. Three similar sets of HOMOs and LUMOs accounted for the second main excitonic feature for both the [Au_13_–Cl_2_] and [Au_13_–Br_2_] clusters, consistent with the similar PLE and PL spectra for these two clusters. For the [Au_13_–I_2_] cluster, five sets of HOMOs and LUMOs were found to be involved in the second excitonic peak. However, not all these transitions might have ended up in radiative recombination, which caused the lower PL intensity in the I-coordinated clusters.

## 3. Conclusion

In summary, we have synthesized three halogen-ligated Au_13_ clusters [Au_13_–X_2_] (X = Cl, Br, and I) and investigated the effects of the halogen ligands on the electronic structures and photophysical properties of the clusters. The icosahedral Au_13_ core structure is preserved during the ligand exchange reactions from Cl to Br to I. The HOMO–LUMO gaps of the clusters are slightly decreased, as well as the PL intensities along the same direction. Theoretical studies indicated that these clusters all have similar electronic structures. The HOMOs of the clusters are mainly from halogen p orbitals and the LUMOs are mainly on the Au_13_ core. The different absorption features in the I-coordinated cluster are understood based on the electronic structure calculations. We have found that different halogens have some subtle effects on the electronic structures and photophysical properties of the clusters due to the different Au–X interactions.

## 4. Experimental

Synthesis of [Au_13_(dppe)_5_X_2_]X_3_: see Section S1 in the ESI.†

### 4.1 Chemicals and preparations

[1,2-Bis(diphenylphosphino)ethane]dichlorodigold(i) (Au_2_(dppe)Cl_2_, 96%), hydrochloric acid (HCl, 37%), hydrobromic acid (HBr, 48%), hydroiodic acid (HI, 57%) and other chemicals were obtained from Sigma-Aldrich and were used as received unless mentioned otherwise. All the solvents were of technical grade. All glassware was washed with aqua regia and cleaned thoroughly before use.

### 4.2 Characterization

NMR spectra were recorded on a 400 MHz Bruker UltraShield spectrometer using MeOD as the solvent. Chemical shifts were reported in ppm and were referenced to tetramethylsilane (internal) for ^1^H and 85% H_3_PO_4_ (external) for ^31^P-NMR. Unless mentioned otherwise, all the NMR spectra were measured at 298 K. The mass spectra were measured in the positive ion mode. A methanol solution of AuNCs (≈0.5 mg mL^−1^) was introduced into an Agilent 6530 Accurate Mass Q-TOF LC-MS system (with Agilent 1260 HPLC) *via* flow injection. The ESI mobile phase was made of acetonitrile/water (50/50) with ≈0.05% formic acid at a flow rate of 200 μL min^−1^. The gas temperature of the ESI source was 130 °C at a flow rate of 8 L min^−1^. The fragmentor voltage was set at 50 V, skimmer at 65 V, and the capillary voltage (*V*_cap_) at 3500 V. The mass range measured was up to 20 000 for MS. The assignments were based on high resolution *m*/*z* values and isotopic distributions. UV-Vis-NIR spectra were measured in methanol solution of [Au_13_–X_2_] on an Agilent Technologies Cary 5000 UV-Vis spectrophotometer. The solution PL and QY measurements were performed on an Edinburgh Instruments FS5 fluorescence spectrometer. [Au_13_–X_2_] clusters were dissolved in methanol for measurements. The PLQYs were measured by using an FS5 Spectrometer with a built-in integrating sphere.

### 4.3 Computational methods

The structures of [Au_13_–X_2_]^3+^ (X = Cl, Br, and I) were optimized based on the crystal structure reported previously using the B3PW91 hybrid density functional. The 6-31G(d) basis set was employed for C, H, and P, and the LANL2DZ basis set was employed for Au. The harmonic vibrational frequencies were also calculated to confirm that the optimized structures are global minima with no imaginary frequencies. Time-dependent DFT (TD-DFT) calculations were conducted to obtain the simulated UV-vis absorption spectra. All the calculations were carried out using the Gaussian 09 package.^54^

## Conflicts of interest

There are no conflicts to declare.

## Supplementary Material

NA-002-D0NA00662A-s001

## References

[cit1] Daniel M.-C., Astruc D. (2004). Chem. Rev..

[cit2] Jin R., Zeng C., Zhou M., Chen Y. (2016). Chem. Rev..

[cit3] Shichibu Y., Kamei Y., Konishi K. (2012). Chem. Commun..

[cit4] Qu X., Li Y., Li L., Wang Y., Liang J., Liang J. (2015). J. Nanomater..

[cit5] Wu Z., Jin R. (2010). Nano Lett..

[cit6] Pyo K., Thanthirige V. D., Kwak K., Pandurangan P., Ramakrishna G., Lee D. (2015). J. Am. Chem. Soc..

[cit7] Yamazoe S., Koyasu K., Tsukuda T. (2014). Acc. Chem. Res..

[cit8] Zhang Y., Song P., Chen T., Liu X., Chen T., Wu Z., Wang Y., Xie J., Xu W. (2018). Proc. Natl. Acad. Sci. U. S. A..

[cit9] De Silva N., Ha J.-M., Solovyov A., Nigra M. M., Ogino I., Yeh S. W., Durkin K. A., Katz A. (2010). Nat. Chem..

[cit10] Valden M., Lai X., Goodman D. W. (1998). Science.

[cit11] Giljohann D. A., Seferos D. S., Daniel W. L., Massich M. D., Patel P. C., Mirkin C. A. (2010). Angew. Chem., Int. Ed..

[cit12] Yougbare S., Chang T.-K., Tan S.-H., Kuo J.-C., Hsu P.-H., Su C.-Y., Kuo T.-R. (2019). Int. J. Mol. Sci..

[cit13] Jadzinsky P. D., Calero G., Ackerson C. J., Bushnell D. A., Kornberg R. D. (2007). Science.

[cit14] Yan N., Xia N., Liao L., Zhu M., Jin F., Jin R., Wu Z. (2018). Sci. Adv..

[cit15] Kang X., Zhu M. (2019). Chem. Soc. Rev..

[cit16] Zhang J., Li Z., Zheng K., Li G. (2018). Phys. Sci. Rev..

[cit17] Sugiuchi M., Maeba J., Okubo N., Iwamura M., Nozaki K., Konishi K. (2017). J. Am. Chem. Soc..

[cit18] Kamei Y., Shichibu Y., Konishi K. (2011). Angew. Chem., Int. Ed..

[cit19] Yao L.-Y., Yam V. W.-W. (2016). J. Am. Chem. Soc..

[cit20] Tomihara R., Hirata K., Yamamoto H., Takano S., Koyasu K., Tsukuda T. (2018). ACS Omega.

[cit21] Narouz M. R., Osten K. M., Unsworth P. J., Man R. W., Salorinne K., Takano S., Tomihara R., Kaappa S., Malola S., Dinh C.-T. (2019). Nat. Chem..

[cit22] Briant C. E., Theobald B. R., White J. W., Bell L. K., Mingos D. M. P., Welch A. J. (1981). J. Chem. Soc., Chem. Commun..

[cit23] Jin S., Wang S., Zhu M. (2019). Chem.–Asian J..

[cit24] Shichibu Y., Suzuki K., Konishi K. (2012). Nanoscale.

[cit25] Shichibu Y., Konishi K. (2010). Small.

[cit26] Zhang S.-S., Feng L., Senanayake R. D., Aikens C. M., Wang X.-P., Zhao Q.-Q., Tung C.-H., Sun D. (2018). Chem. Sci..

[cit27] Jin S., Du W., Wang S., Kang X., Chen M., Hu D., Chen S., Zou X., Sun G., Zhu M. (2017). Inorg. Chem..

[cit28] Zhang S.-S., Senanayake R. D., Zhao Q.-Q., Su H.-F., Aikens C. M., Wang X.-P., Tung C.-H., Sun D., Zheng L.-S. (2019). Dalton Trans..

[cit29] Chen J., Zhang Q.-F., Williard P. G., Wang L.-S. (2014). Inorg. Chem..

[cit30] Zhang H.-F., Stender M., Zhang R., Wang C., Li J., Wang L.-S. (2004). J. Phys. Chem. B.

[cit31] Chen J., Zhang Q.-F., Bonaccorso T. A., Williard P. G., Wang L.-S. (2014). J. Am. Chem. Soc..

[cit32] Zhang Q. F., Williard P. G., Wang L. S. (2016). Small.

[cit33] Guan Z.-J., Hu F., Li J.-J., Wen Z.-R., Lin Y.-M., Wang Q.-M. (2020). J. Am. Chem. Soc..

[cit34] Das A., Li T., Nobusada K., Zeng Q., Rosi N. L., Jin R. (2012). J. Am. Chem. Soc..

[cit35] Li J. J., Guan Z. J., Lei Z., Hu F., Wang Q. M. (2019). Angew. Chem., Int. Ed..

[cit36] Zhu M., Lanni E., Garg N., Bier M. E., Jin R. (2008). J. Am. Chem. Soc..

[cit37] Kenzler S., Fetzer F., Schrenk C., Pollard N., Frojd A. R., Clayborne A. Z., Schnepf A. (2019). Angew. Chem., Int. Ed..

[cit38] Jin R., Liu C., Zhao S., Das A., Xing H., Gayathri C., Xing Y., Rosi N. L., Gil R. R., Jin R. (2015). ACS Nano.

[cit39] Liao L., Zhuang S., Yao C., Yan N., Chen J., Wang C., Xia N., Liu X., Li M.-B., Li L. (2016). J. Am. Chem. Soc..

[cit40] Zeng C., Chen Y., Liu C., Nobusada K., Rosi N. L., Jin R. (2015). Sci. Adv..

[cit41] Schmid G. (2008). Chem. Soc. Rev..

[cit42] Narouz M. R., Takano S., Lummis P. A., Levchenko T. I., Nazemi A., Kaappa S., Malola S., Yousefalizadeh G., Calhoun L. A., Stamplecoskie K. G. (2019). J. Am. Chem. Soc..

[cit43] Weerawardene K. D. M., Pandeya P., Zhou M., Chen Y., Jin R., Aikens C. M. (2019). J. Am. Chem. Soc..

[cit44] Xiong X.-G., Wang Y.-L., Xu C.-Q., Qiu Y.-H., Wang L.-S., Li J. (2015). Dalton Trans..

[cit45] Liu H.-T., Xiong X.-G., Dau P. D., Wang Y.-L., Li J., Wang L.-S. (2011). Chem. Sci..

[cit46] Rai A., Singh A., Ahmad A., Sastry M. (2006). Langmuir.

[cit47] Zhang J., Langille M. R., Personick M. L., Zhang K., Li S., Mirkin C. A. (2010). J. Am. Chem. Soc..

[cit48] Himstedt R., Hinrichs D., Sann J., Weller A., Steinhauser G., Dorfs D. (2019). Nanoscale.

[cit49] Akkerman Q. A., D'Innocenzo V., Accornero S., Scarpellini A., Petrozza A., Prato M., Manna L. (2015). J. Am. Chem. Soc..

[cit50] Shamsi J., Urban A. S., Imran M., De Trizio L., Manna L. (2019). Chem. Rev..

[cit51] Zeng J.-L., Guan Z.-J., Du Y., Nan Z.-A., Lin Y.-M., Wang Q.-M. (2016). J. Am. Chem. Soc..

[cit52] Protesescu L., Yakunin S., Bodnarchuk M. I., Krieg F., Caputo R., Hendon C. H., Yang R. X., Walsh A., Kovalenko M. V. (2015). Nano Lett..

[cit53] Zhang J., Zhou Y., Zheng K., Abroshan H., Kauffman D. R., Sun J., Li G. (2018). Nano Res..

[cit54] Frisch M. J. , Trucks G. W., Schlegel H. B., Scuseria G. E., Robb M. A., Cheeseman J. R., Scalmani G., Barone V., Mennucci B., Petersson G. A., Nakatsuji H., Caricato M., Li X., Hratchian H. P., Izmaylov A. F., Bloino J., Zheng G., Sonnenberg J. L., Hada M., Ehara M., Toyota K., Fukuda R., Hasegawa J., Ishida M., Nakajima T., Honda Y., Kitao O., Nakai H., Vreven T., Montgomery Jr J. A., Peralta J. E., Ogliaro F., Bearpark M., Heyd J. J., Brothers E., Kudin K. N., Staroverov V. N., Keith T., Kobayashi R., Normand J., Raghavachari K., Rendell A., Burant J. C., Iyengar S. S., Tomasi J., Cossi M., Rega N., Millam J. M., Klene M., Knox J. E., Cross J. B., Bakken V., Adamo C., Jaramillo J., Gomperts R., Stratmann R. E., Yazyev O., Austin A. J., Cammi R., Pomelli C., Ochterski J. W., Martin R. L., Morokuma K., Zakrzewski V. G., Voth G. A., Salvador P., Dannenberg J. J., Dapprich S., Daniels A. D., Farkas O., Foresman J. B., Ortiz J. V., Cioslowski J. and Fox D. J., Gaussian 09, Revision D.01, Gaussian, Inc., Wallingford CT, 2013

