# Supplementary material for: Halogen effects on the electronic and optical properties of Au13 nanoclusters
Source: Nanoscale Adv. 2020 Aug 31;2(10):4902–7. doi: 10.1039/d0na00662a (PMC9419307; doi:10.1039/d0na00662a)
Supplement: NA-002-D0NA00662A-s001 [file NA-002-D0NA00662A-s001.pdf]

Electronic Supplementary Material (ESI) for Nanoscale Advances.
This journal is © The Royal Society of Chemistry 2020

Supporting Information

The Halogen Effects on the Electronic and Optical Properties of the Au13 Nanocluster

Ze-Hua Gao,[†a] Jia Dong,[†a] Qian-Fan Zhang,[a] and Lai-Sheng Wang[*a]

[a] Department of Chemistry, Brown University, Providence, RI 02912, USA.

[†] These authors contributed equally to this work.

Corresponding Author E-mail: E-mail: lai-sheng_wang@brown.edu

Index:

Figure S1: High resolution ESI-MS of [Au_{13}-X_2] clusters.

Figure S2-5: ^{1}H-NMR spectra of [Au_{13}-X_2] clusters.

Figure S6: Temperature-dependent UV-Vis spectra of [Au_{13}-X_2].

Figure S7: Thermal stability of [Au_{13}-X_2] at 50 ^oC.

Figure S8-10: Optical energy gap determination on [Au_{13}-X_2] clusters.

Figure S11: Comparison of the theoretical absorption spectra and photoluminescent excitation spectra.

Figure S12-14: Composition of the second excitonic peaks of [Au_{13}-X_2] clusters.

Table S1: Structure properties of the simulated [Au_{13}-X_2] clusters.

S1: Synthesis of $[Au_{13}(dppe)_5X_2]X_3$

Direct synthesis of $[Au_{13}(dppe)_5X_2]X_3$: The method is divided into two steps.[1] Step 1: $Au_2(dppe)Cl_2$ (30.2 mg, 0.035 mmol) was dissolved in 24 mL dichloromethane in a 50 mL flask, 1mL suspended $NaBH_4$ solution (33.2 mg fresh $NaBH_4$ in 5mL ethanol) was rapidly injected to the flask under 900 rpm stirring. The mixture was stirred at room temperature for 3h. After that, the solvent was removed by rotary evaporator and the residue was re-suspended in dichloromethane and centrifuged to remove insoluble precipitates. The solute was then evaporated to dryness to give a dark-brown solid. Step 2: The dark-brown solid was dissolved in 5 mL ethanol to give deep red color solution. 0.1 mL 12 M HCl (or 0.14 mL 8.9 M HBr, or 0.16 mL 7.57 M HI) was added to the solution at the speed of 5 seconds per drop. The vial was then shaded from light by aluminum foil covering, especially for $[Au_{13}\text{-}I_2]$, and stirred at room temperature for 24 h. After that, the volatiles were evaporated and the residue was washed with acetone, dried again, suspended in ethanol and centrifuged to remove precipitates. Red (deep red for HBr and brown for HI) solids were obtained after evaporation and the total yield was nearly 30% based on the gold precursor. The solvent wash process could be repeated if the final product is not pure enough.

$[Au_{13}\text{-}X_2]$ (X=Br, I) were also obtained through a ligand exchange method: By adding excess amounts (typically with a ratio of Br/Cl or I/Cl equal to three) of potassium bromide (KBr) (2.8 mg, 0.024 mmol) or potassium iodide (KI) (3.9 mg, 0.024 mmol) to 5 mL methanol solution of $[Au_{13}\text{-}Cl_2]$ (20 mg, 0.004 mmol), the color immediately turned from red to deep red (Br) or brown (I), respectively. After the evaporation of the volatiles, products were purified by acetone and methanol as in the step 2 of the direct method. The yield was nearly 60% for both. $[Au_{13}\text{-}I_2]$ could be produced by introducing KI to $[Au_{13}\text{-}Br_2]$ similarly. The reverse reactions were not successful. Similar experiment using KF was also performed, Au_{13} clusters decomposed instead of forming $[Au_{13}\text{-}F_2]$.

S2. High Resolution ESI-MS of [Au_{13}-X_2]

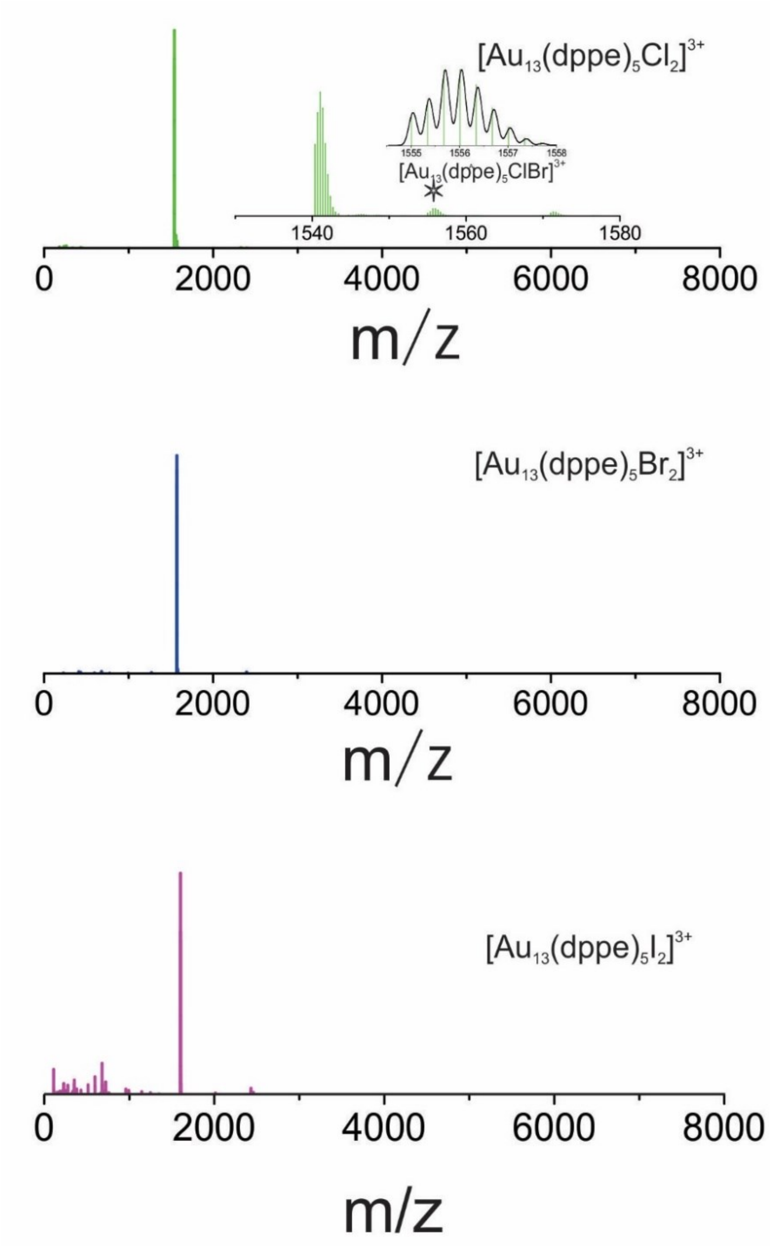

Figure S1: ESI-MS spectrum of [Au_{13}-X_2] clusters in the range of 0 ~ 8000 m/z. Green: [Au_{13}-Cl_2], the inserted star corresponds to [$Au_{13}(dppe)_5ClBr]^{3+}$. Blue: [$Au_{13}$-$Br_2$]. Purple: [$Au_{13}$-$I_2$].

S3.¹H-NMR spectrum of [Au_{13}-X_2]

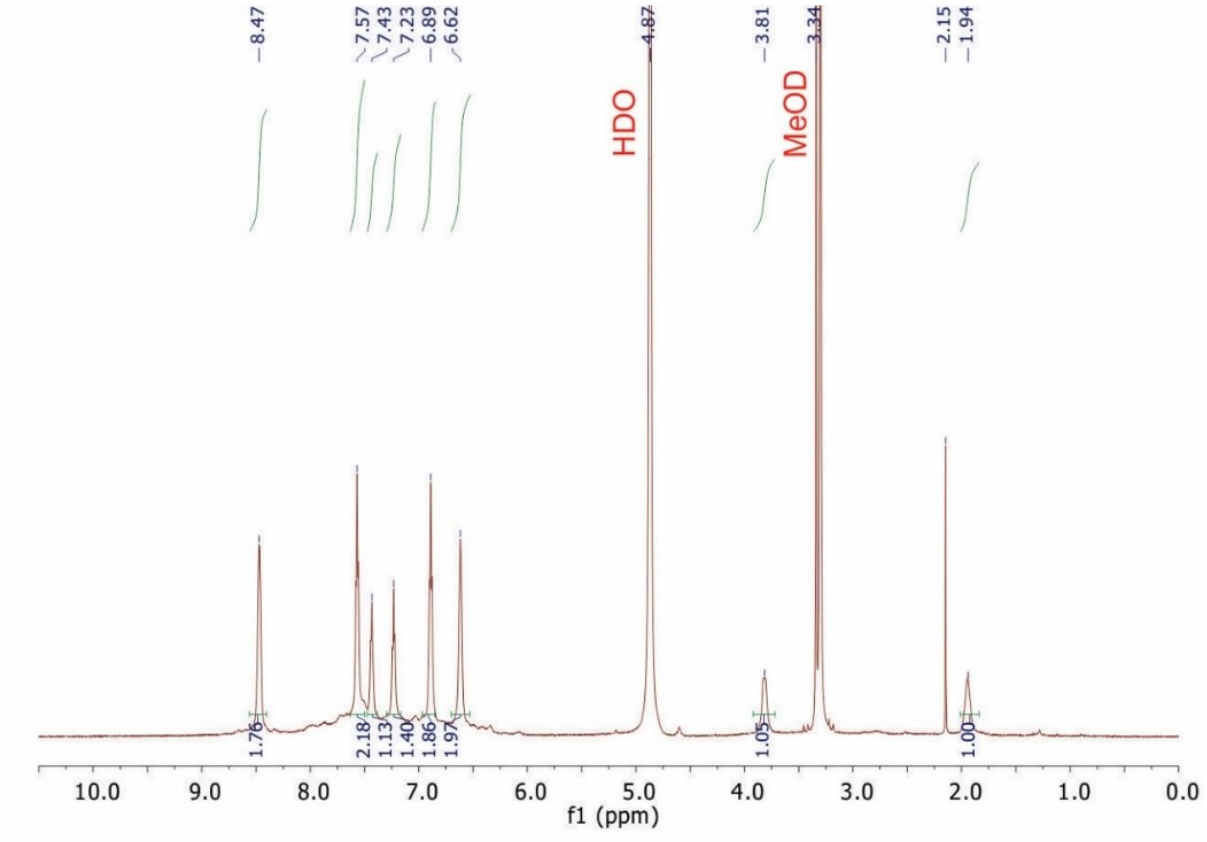

Figure S2: ^{1}H-NMR spectrum of [Au_{13}-Cl_2] (MeOD, 400MHz).

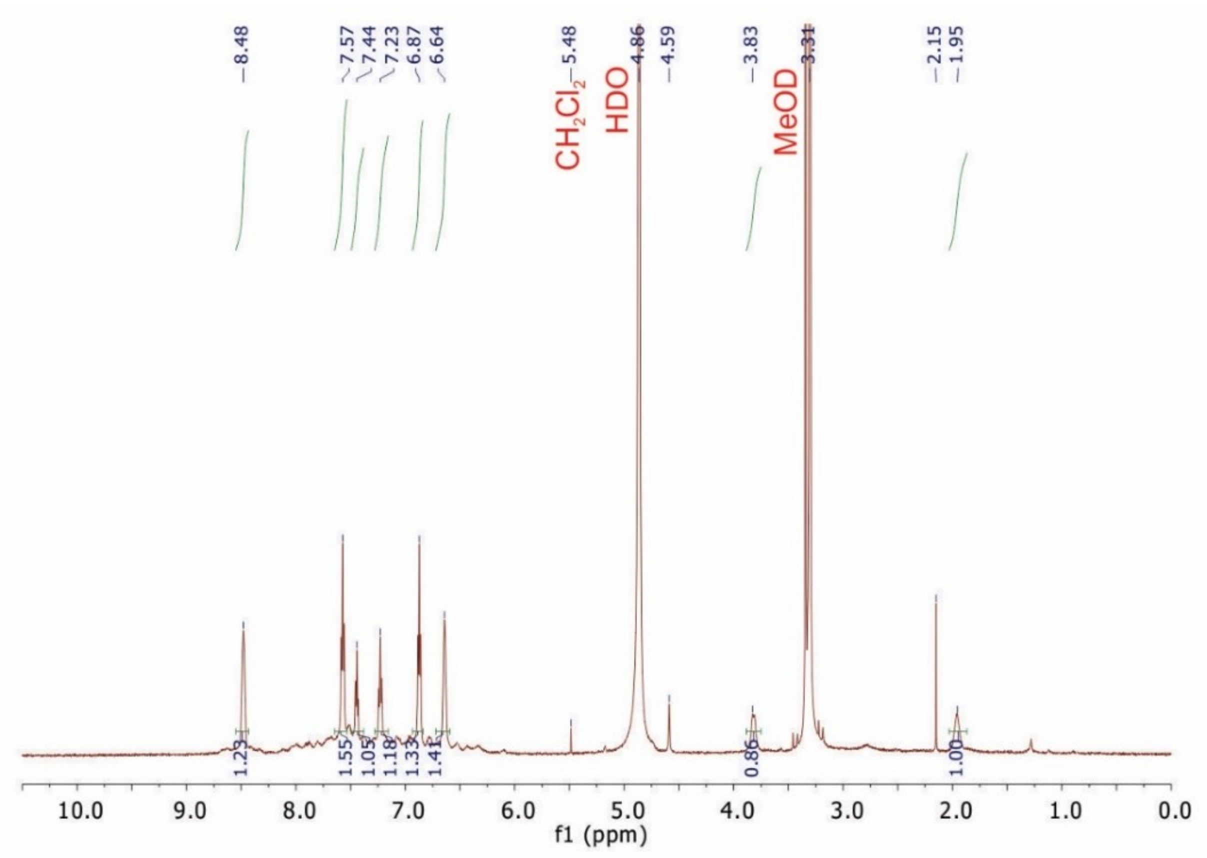

Figure S3: ^{1}H-NMR spectrum of [Au_{13}-Br_2] (MeOD, 400MHz).

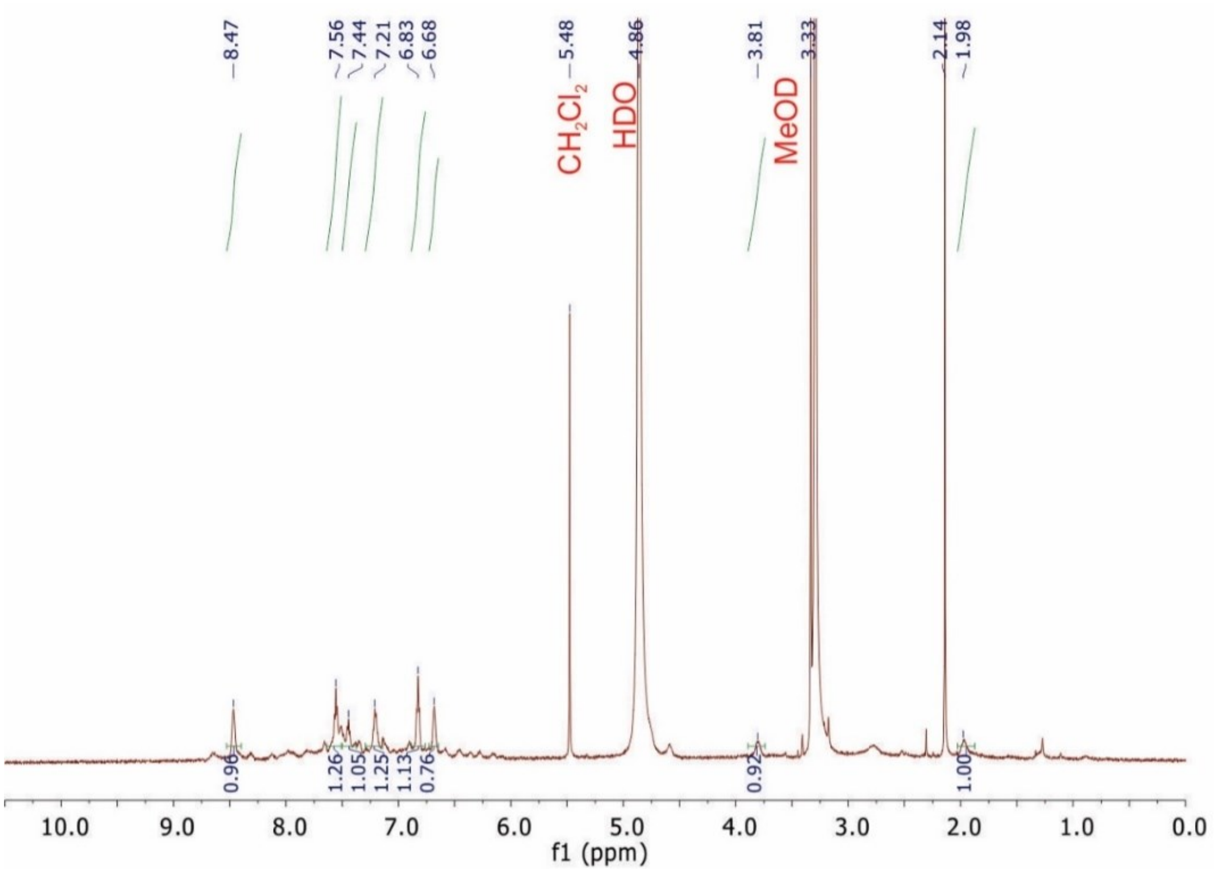

Figure S4: ^{1}H-NMR spectrum of [Au_{13}-I_2] (MeOD, 400MHz).

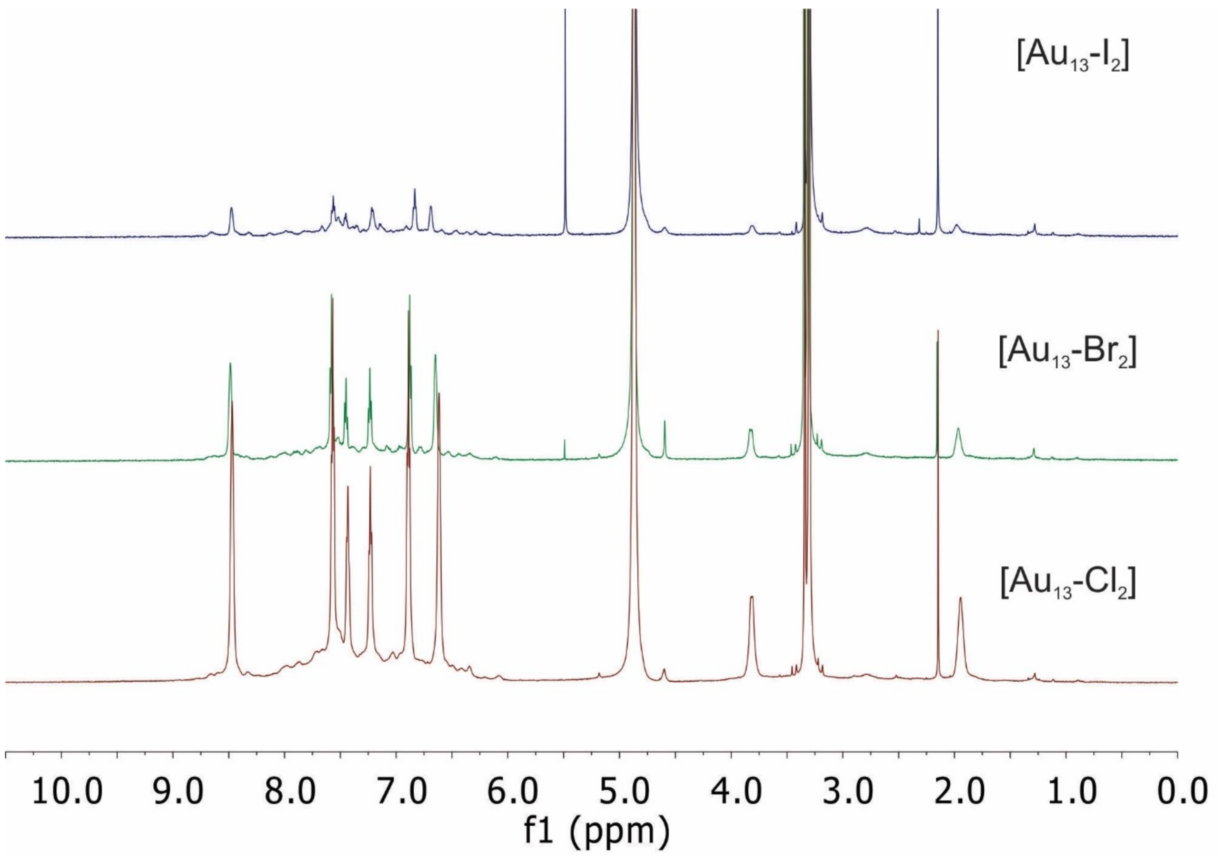

Figure S5: Compiled ^{1}H-NMR spectrum of [Au_{13}-X_2].

S4. Temperature-dependent UV-Vis spectra of [Au_{13}-X_2].

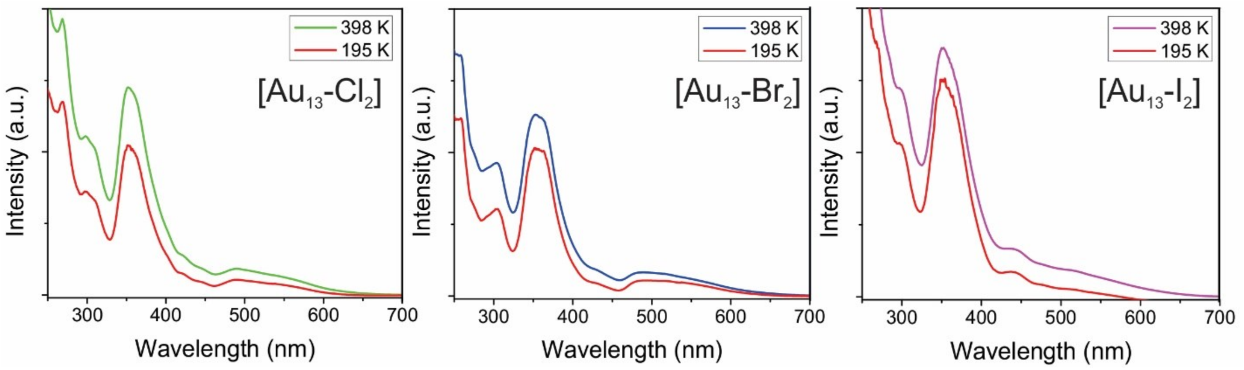

Figure S6: Temperature-dependent UV-Vis spectra of [Au_{13}-X_2]. From left to right: [Au_{13}-Cl_2], [Au_{13}-Br_2], [Au_{13}-I_2].

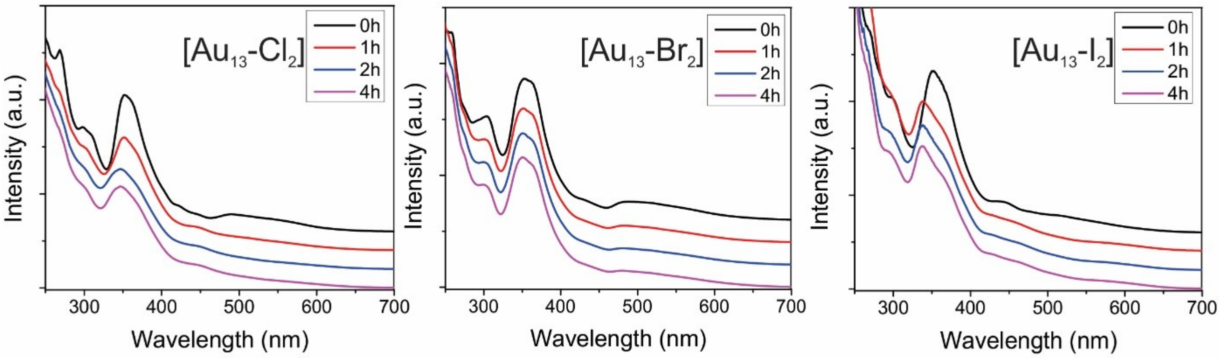

S5. Thermal stability measurements of [Au_{13}-X_2].

Figure S7: Thermal stability of [Au_{13}-X_2] at 50 ^{o}C. From left to right: [Au_{13}-Cl_2], [Au_{13}-Br_2], [Au_{13}-I_2].

S6. Tauc plot for [Au_{13}-X_2]

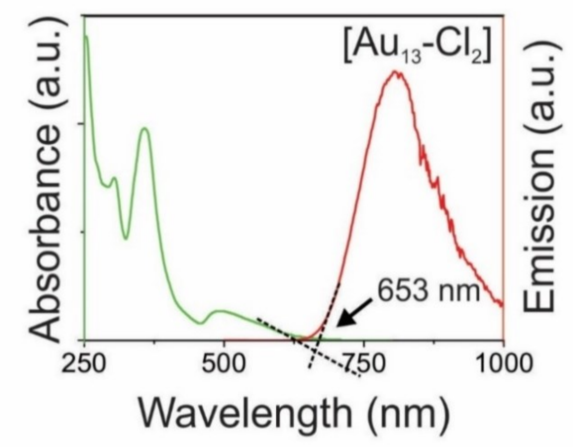

Figure S8: Tauc plot for [Au_{13}-Cl_2].

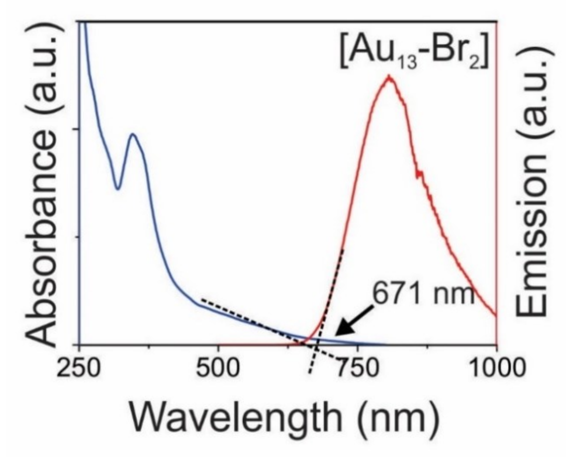

Figure S9: Tauc plot for [Au_{13}-Br_2].

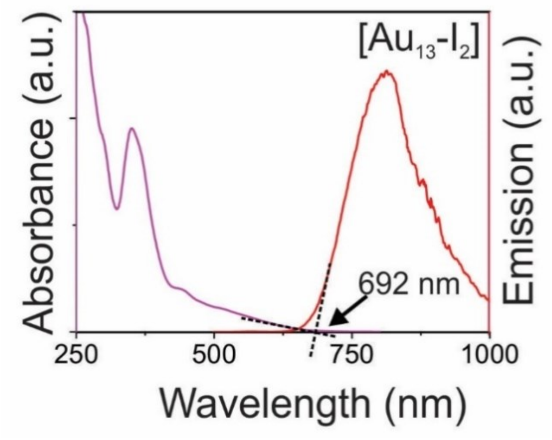

Figure S10: Tauc plot for [Au_{13}-I_2].

S7. Computational results of [Au_{13}-X_2]

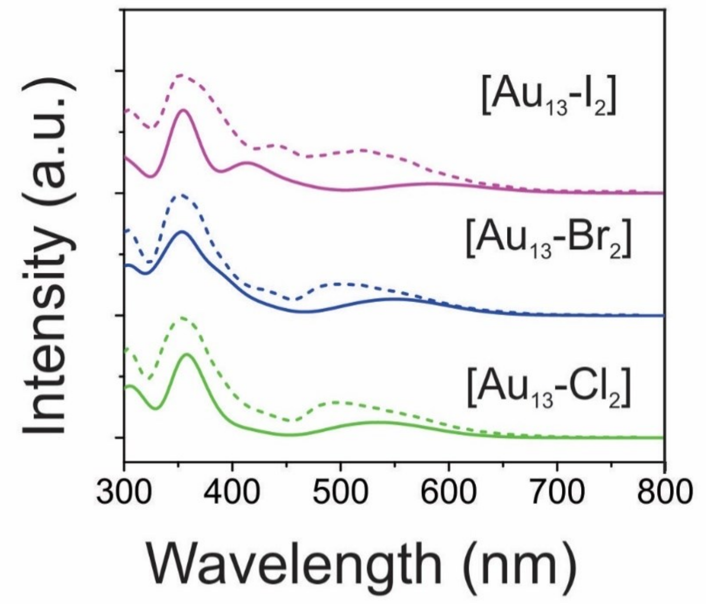

Figure S11. Comparison of theoretical absorption spectra (solid lines) and experimental PLE spectra (dotted lines).

Table S1. Structure properties of the simulated [Au_{13}-X_2] clusters.

[Au_{13}-X_2]	Au-X (Å)	Central Au-Au-X angle (°)	Central Au-Au (Å)	Average Au-Au (Å)
Cl	2.335	180.0	2.841	2.948
Br	2.435	180.0	2.842	2.949
I	2.629	179.1	2.843	2.949

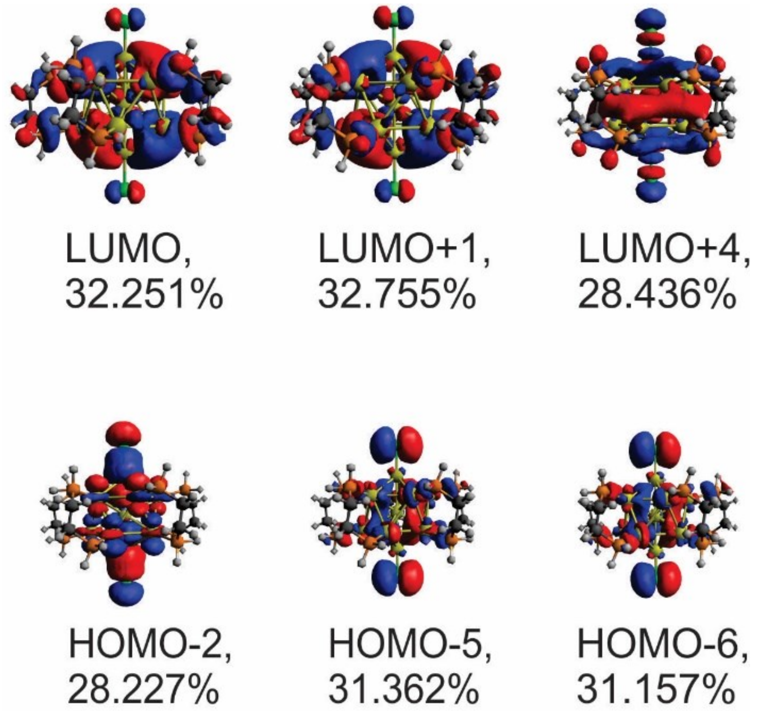

Figure S12: Compositions of the second excitonic peak for [Au_{13}-Cl_2] with contribution larger than 5%.

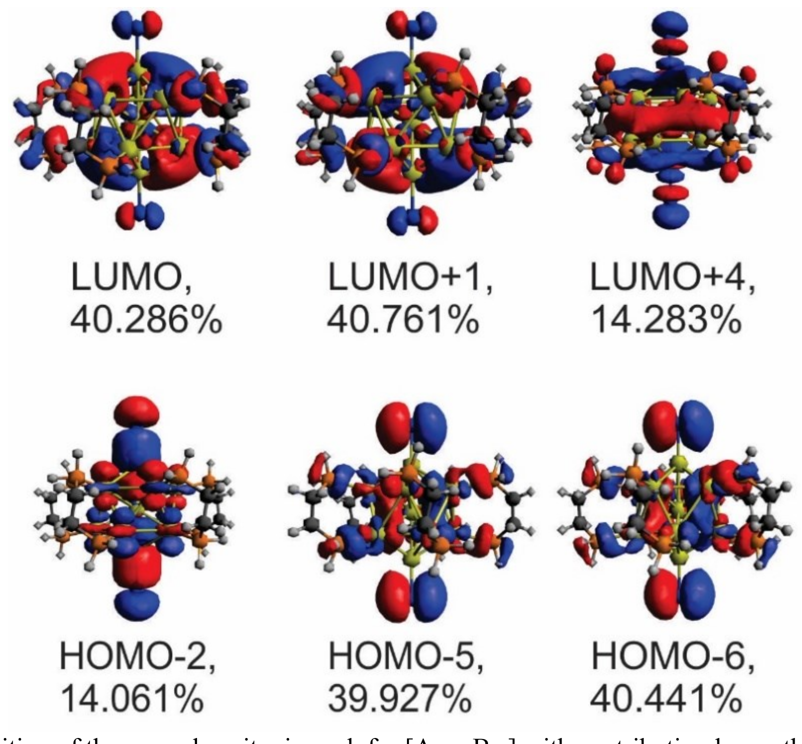

Figure S13: Composition of the second excitonic peak for [Au_{13}-Br_2] with contribution larger than 5%.

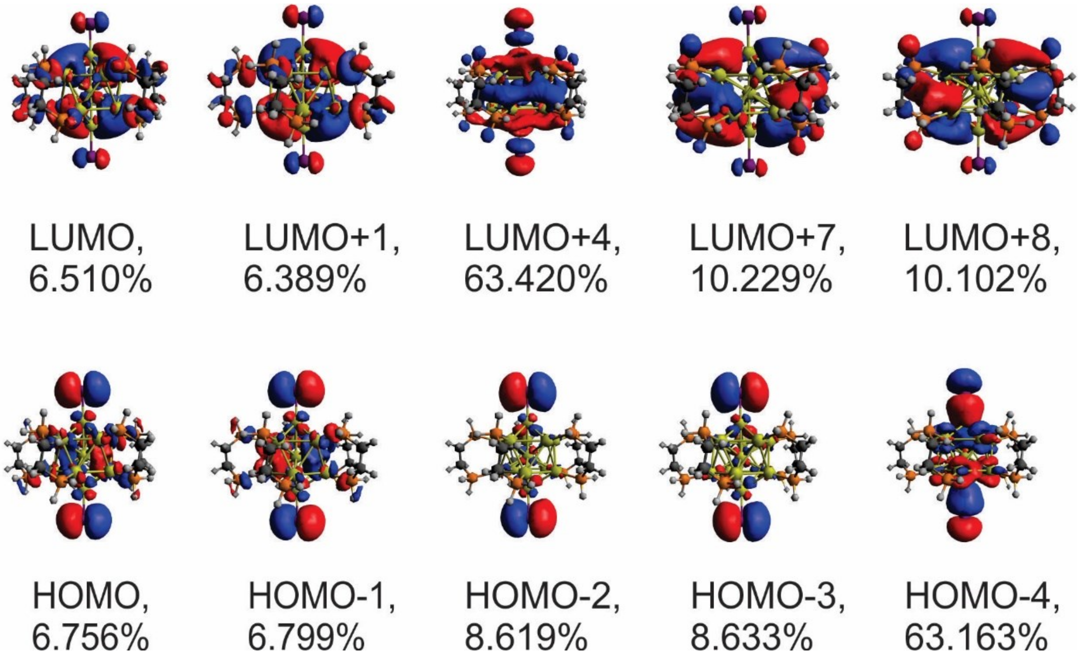

Figure S14: Composition of the second excitonic peak for [Au_{13}-I_2] with contribution larger than 5%.

1. Y. Shichibu and K. Konishi, *Small*, 2010, **6**, 1216-1220.
